# Can malocclusion provide clinicians with information for differential diagnosis of temporomandibular joint diseases?: A review

**DOI:** 10.1097/MD.0000000000029247

**Published:** 2022-08-19

**Authors:** Shinpei Matsuda, Taihiko Yamaguchi, Saki Mikami, Hitoshi Yoshimura, Akihito Gotouda

**Affiliations:** a Division of Dentistry and Oral Surgery, Department of Sensory and Locomotor Medicine, Faculty of Medical Sciences, University of Fukui, Fukui, Japan; b Division of Oral Functional Science, Department of Crown and Bridge Prosthodontics, Graduate School of Dental Medicine, Hokkaido University, Hokkaido, Japan; c Department of Temporomandibular Disorders, Center for Advanced Oral Medicine, Hokkaido University Hospital, Hokkaido, Japan.

**Keywords:** diagnostic tree, differential diagnosis, malocclusion, temporomandibular joint

## Abstract

The aim of this literature review was to summarize the clinical characteristics and symptoms of temporomandibular joint diseases, and to discuss the associations between temporomandibular joint diseases and categorization of malocclusion. Electronic literature searches were performed using the PubMed database. The authors established a differential diagnostic method for temporomandibular joint diseases related to malocclusion. A literature search using PubMed yielded 213 texts, of which based on exclusion criteria, 28 were included in this study. Malocclusions were categorized into 5 types. The authors suggested a diagnostic tree of temporomandibular joint diseases based on the types of malocclusion and 4 variables in clinical characteristics and symptoms. Clinicians treating malocclusions must attempt to clarify the cause of the occlusal condition. If caused by temporomandibular joint disease, it is important to make a proper differential diagnosis at first, and not to overlook the causative disease. Further clinical knowledge of associations between temporomandibular joint diseases and malocclusions should be accumulated, and the diagnostic tree should be improved based on new information.

## 1. Introduction

Malocclusion can be interpreted as an irregular dental arch and/or occlusion associated with congenital and acquired factors, including harmful oral habits such as digit sucking, tongue thrust, lip habit, habitual mouth breathing, and unilateral mastication habit.^[1,2]^ The term malocclusion indicates deep overbite, midline deviation, excessive overjet, crossbite, malalignment, space, and open bite.^[2]^ It is the third most prevalent oral disease after tooth decay and periodontal disease. Unilateral cases are more frequent than bilateral cases.^[3,4]^ It may affect esthetic appearance, occlusal function, psychosocial well-being, and health-related quality of life. Malocclusion is also defined by the World Health Organization as a handicapping dentofacial anomaly.^[1,2]^

Temporomandibular joint (TMJ) diseases, including temporomandibular disorders (TMD), are closely related to malocclusion.^[3,5]^ Acute lateral and/or vertical malocclusion is caused by effusions in the TMJ associated with trauma, infection, and arthritis.^[3,6,7]^ It has recently become possible to observe joint effusions noninvasively and accurately owing to advances in magnetic resonance imaging (MRI) technology. However, recent studies have suggested that there is no clear association between the pathophysiology of TMD and any specific type of dental occlusion. TMD is generally accepted as a multifactorial disease.^[8–11]^ However, a study based on MRI of patients with anterior open bite suggested that TMJ degeneration associated with displaced discs caused the development of acquired anterior open bite.^[12]^ Furthermore, the authors’ previous study suggested that TMJ closed lock, as a condition of TMD, and related acquired anterior open bite might occur regardless of whether an occlusal splint had been used.^[13]^ The cause-and-effect relationship between TMD and dental occlusion is controversial topic even today. It needs to be discussed from the perspectives of both etiologies and results.^[5]^ Additionally, the authors report on cases of malocclusion associated with metastases to the TMJ. They suggest that general dentists and oral and maxillofacial surgeons should rule out the presence of malignant diseases at first by imaging examinations in cases of malocclusion that cannot easily be repositioned.^[14]^ The authors also report a rare case of malocclusion associated with trigeminal neuropathy caused by maxillofacial trauma.^[15]^ Malocclusions are caused by various TMJ diseases and conditions. These findings may be useful in the early differential diagnosis of TMJ diseases and conditions, and might provide important information about management and treatment methods for general dentists and oral and maxillofacial surgeons. There are no detailed reports on the association between TMJ diseases and types of malocclusion. Furthermore, there has been no literature review discussing their usefulness in the assessment of malocclusion in the differential diagnosis of TMJ diseases. The authors consider it important to summarize the clinical characteristics and symptoms of TMJ diseases, and to establish a differential diagnostic method for TMJ diseases, such as a diagnostic tree, for clinicians treating malocclusion.

The aim of this literature review was to summarize the clinical characteristics and symptoms of TMJ diseases, including malocclusion, and to discuss the associations between TMJ diseases and categorization of malocclusion. Based on these associations, the authors established a differential diagnostic method, namely a diagnostic tree, of TMJ diseases based on the types of malocclusion.

## 2. Methods

### 2.1. Literature search strategy

Electronic searches of PubMed (National Center for Biotechnology Information, NCBI) were performed on April 20, 2020. Literature search strategy was as follows: ((((((((malocclusion[Title/Abstract]) OR occlusal abnormality[Title/Abstract]) OR abnormal occlusion[Title/Abstract]) AND temporomandibular joint[MeSH Terms]) AND English[Language]) NOT orthodontic[MeSH Terms]) NOT orthognathic[MeSH Terms]) NOT postoperative[MeSH Terms]) AND (“1970”[Date - Publication]: “2019”[Date - Publication]).

### 2.2. Selection of studies and data extraction

The authors’ literature search initially included case reports, case series, and narrative reviews where the full text was available. The literature was evaluated by 2 reviewers (SM and SM) and relevant texts were extracted.

The exclusion criteria were as follows: cases of only subjective occlusal symptoms without obvious identifiable occlusal disharmony, controlled clinical trials, cross-sectional studies, systematic reviews, and letters to editors; experimental or animal trials; reports of malocclusion after orthodontic treatment and/or dental treatment, including occlusal splints and reports of postoperative malocclusion; and literature for which the full text was unavailable.

Clinical characteristics and symptoms of TMJ diseases, including the types of malocclusions, were extracted. The authors summarized the following points: type of malocclusion involving side, acute or chronic occlusal change, pain, inflammation, swelling, and limitation of mouth opening. Methods for malocclusion diagnosis, treatment, and management were not extracted.

## 3. Results

By performing an electronic literature search using PubMed, 213 texts were extracted. These texts were assessed for eligibility, and 28 eligible texts were included in this study. The diseases and conditions include TMD, septic arthritis, condylar hyperplasia, dislocation, idiopathic condylar resorption, juvenile idiopathic arthritis, rheumatoid arthritis, condylar fracture, articular disc fracture, ankylosis, chromosomal and genetic abnormalities, or congenital anomalies.

**Figure 1.**
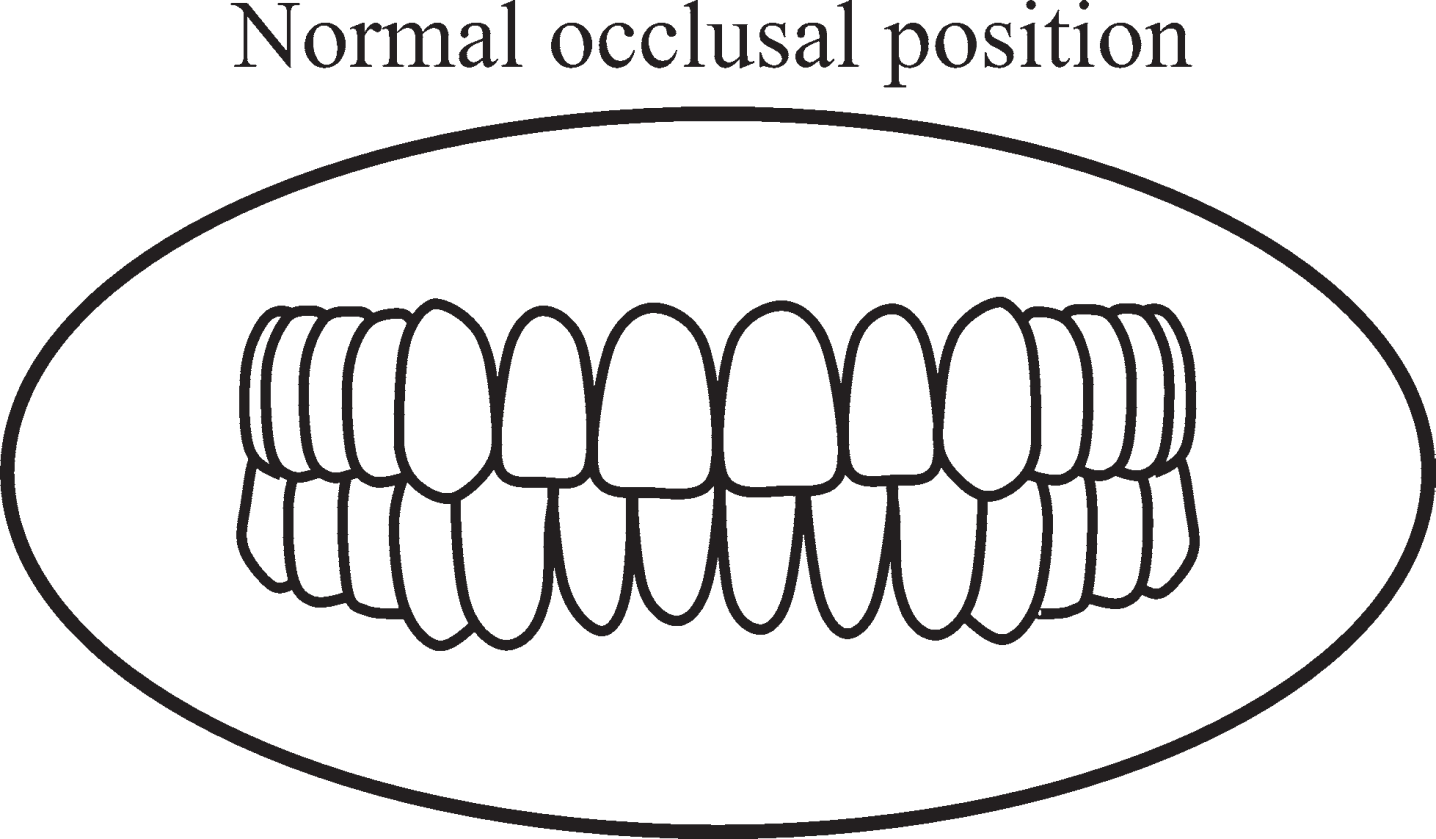
The normal occlusal condition without any diseases.

**Figure 2.**
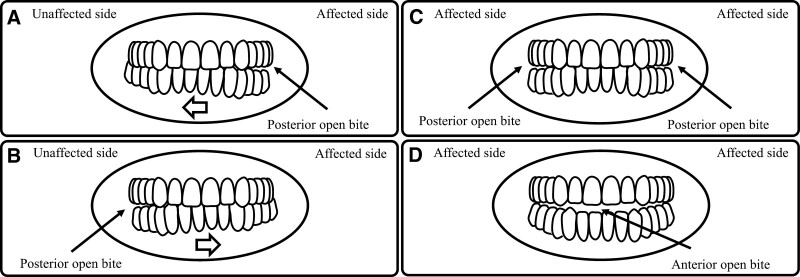
The 5 types of malocclusion. (A) The posterior open bite on the affected side and/or mandibular shift toward the unaffected side. (B) The posterior open bite on the unaffected side and/or mandibular shift towards the affected side. (C) The bilateral posterior open bite. (D) The anterior open bite and/or clockwise-rotation of the mandible.

**Figure 3.**
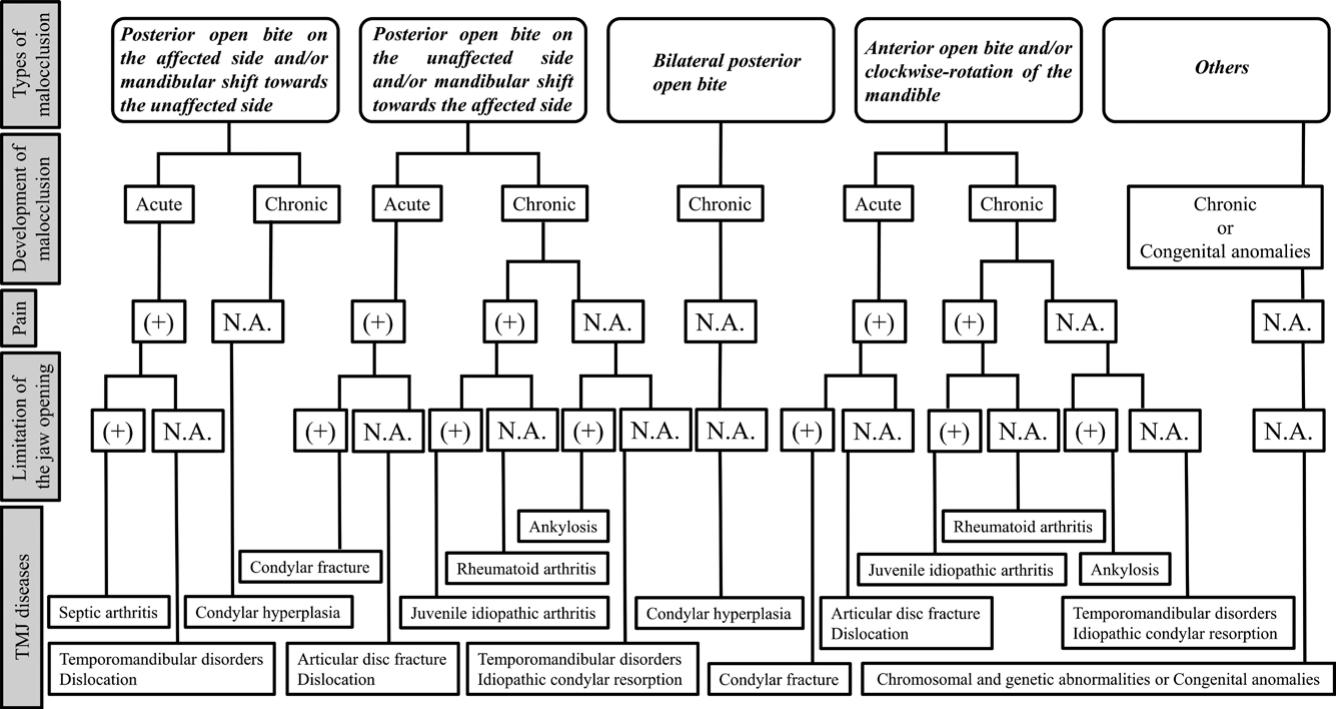
Diagnostic tree of TMJ diseases based on types of malocclusion, and 4 other variables of clinical characteristics and symptoms, TMJ = temporomandibular joint.

The authors categorized malocclusion associated with TMJ diseases into 5 types: posterior open bite on the affected side and/or mandibular shift toward the unaffected side, posterior open bite on the unaffected side and/or mandibular shift toward the affected side; bilateral posterior open bite; anterior open bite and/or clockwise rotation of the mandible; and others (Figs. 1 and 2. Table 1). Duplicate categorization of TMJ diseases into 5 types of malocclusion was allowed in this study. TMD, septic arthritis, condylar hyperplasia, and dislocation were grouped as “Posterior open bite on the affected side and/or mandibular shift toward the unaffected side.” TMD, idiopathic condylar resorption, juvenile idiopathic arthritis, rheumatoid arthritis, condylar fracture, articular disc fracture, ankylosis, and dislocation were grouped as “Posterior open bite on the unaffected side and/or mandibular shift toward the affected side.” Condylar hyperplasia was identified as “bilateral posterior open bite.” TMD, idiopathic condylar resorption, juvenile idiopathic arthritis, rheumatoid arthritis, condylar fracture, articular disc fracture, ankylosis, and dislocation were grouped as “anterior open bite and/or clockwise rotation of the mandible.” Chromosomal and genetic abnormalities or congenital anomalies were grouped as “Others.” Furthermore, the authors created a diagnostic tree of TMJ diseases based on types of malocclusion and 4 variables in clinical characteristics and symptoms as follows: type of malocclusion, acute or chronic occlusal change, presence or absence of pain, and presence or absence of limitation of mouth opening (Fig. 3). The details of the 5 malocclusion types are described below.

**Table 1:** Clinical symptoms of various temporomandibular diseases categorized with types of malocclusion.

Malocclusion										
Temporomandibular diseases	Involving side	Development of malocclusion	Pain	Inflammation	Swelling	Limitation of the mouth opening	Age	Gender	Prevalence	Other remarks
***Posterior open bite on the affected side and/or mandibular sift towards the unaffected side***										
Temporomandibular disorders^[3]^	Unilateral	Acute	(+)	(+)	Not described	Not described	Not described	Not described	Not described	There are bilateral cases.
Septic arthritis^[16]^	Unilateral	Acute	(+)	(+)	(+)	(+)	Not described	Not described	Not described	*S aureus* is the most common pathogen.
Condylar hyperplasia^[17,18]^	Unilateral	Chronic	(+)/(–)	Not described	(-)	(+)/(–)	TYPE 1: Early to middle 20sTYPE 2: Second decade	TYPE 2: Female predominant (3: 1)	Not described	There are bilateral cases. Wolford's classification (4 types)
Dislocation^[19]^	Unilateral	Acute	(+)	Not described	(+)	Not described	Not described	Not described	Not described	Anterior dislocation. There are bilateral cases.
***Posterior open bite on the unaffected side and/or mandibular sift towards the affected side***										
Temporomandibular disorders^[3]^	Unilateral	Chronic	Not described	Not described	Not described	Not described	Not described	Not described	Not described	There are bilateral cases.
Idiopathic condylar resorption^[20–22]^	Unilateral	Chronic	(–)	Not described	(–)	(–)	10–40 years old with a strong predominance for teenagers	Female predominant (9:1)	Not described	There are bilateral cases.
Juvenile idiopathic arthritis^[23–27]^	Unilateral	Chronic	(+)	(+) Inflammation occur in one or more joint	Not described	(+)	Under 16 years old	Girls predominant (3:2)	11–80 per 100,000 childrenMalocclusion observed in 21–69%	There are bilateral cases.
Rheumatoid arthritis^[26]^	Unilateral	Chronic	(+)	(+) Inflammation occur in one or more joint	Not described	Not described	Not described	Not described	Not described	There are bilateral cases.
Condylar fracture ^[28–33]^	Unilateral	Acute	(+)	Not described	Not described	(+)	Mean age: approximately 30 years old	Male predominant	9–49% of all mandibular fractures	There are bilateral cases.
Articular disc fracture^[34]^	Unilateral	Acute	(+)	Not described	Not described	(-)	Not described	Not described	Not described	There are bilateral cases.
Ankylosis^[35–37]^	Unilateral	Chronic	(+) / (-)	Not described	Not described	(+)	Not described	Not described	Not described	There are bilateral cases.
Dislocation^[38]^	Unilateral	Acute	(+)	Not described	Not described	Not described	Pediatric predominant.	Female predominant.	Not described	Upward dislocation.
***Bilateral posterior open bite***										
Condylar hyperplasia^[17,18]^	Bilateral	Chronic	(-)	Not described	Not described	Not described	TYPE 1: Early to middle 20s	Not described	Not described	There are unilateral cases.Only TYPE 1 occur bilaterally.
***Anterior open bite and/or clockwise-rotation of the mandible***										
Temporomandibular disorders^[39]^	Bilateral	Chronic	Not described	Not described	Not described	Not described	Not described	Not described	Not described	
Idiopathic condylar resorption^[20–22]^	Bilateral	Chronic	(–)	Not described	(–)	(–)	10–40 years old with a strong predominance for teenagers	Female predominant (9:1)	Not described	There are unilateral cases.
Juvenile idiopathic arthritis^[23–27]^	Bilateral	Chronic	(+)	(+) Inflammation occur in one or more joint	Not described	(+)	Under 16 years old	Girls predominant (3:2)	11–80/100,000 childrenOcclusal abnormality observed in 21–69%	There are unilateral cases.
Rheumatoid arthritis^[26]^	Bilateral	Chronic	(+)	(+) Inflammation occur in one or more joint	Not described	Not described	Not described	Not described	Not described	There are unilateral cases.
Condylar fracture ^[28–33]^	Bilateral	Acute	(+)	Not described	Not described	(+)	Mean age: approximately 30 years old	Male predominant	9–49% of all mandibular fractures	There are unilateral cases.
Articular disc fracture^[34]^	Bilateral	Acute	(+)	Not described	Not described	(-)	Not described	Not described	Not described	There are unilateral cases.
Ankylosis^[37–39]^	Bilateral	Chronic	(+) / (-)	Not described	Not described	(+)	Not described	Not described	Not described	There are unilateral cases.
Dislocation^[19]^	Bilateral	Acute	(+)	Not described	(+)	Not described	Not described	Not described	Not described	Anterior dislocation. There are unilateral cases.
***Others***										
Chromosomal and genetic abnormalities or congenital anomalies^[40–42]^	Not applicable	Chronic (or congenital anomalies)	Not described	Not described	Not described	Not described	Not described	Not described	Not described	A number of other clinical features

### 3.1. Posterior open bite on the affected side and/or mandibular shift toward the unaffected side

#### 3.1.1. TMD (unilateral case).

Increasing disc space-related thickness of the posterior band is associated with disc displacement with reduction and an inflammatory response of the TMJ component caused by malocclusion (Fig. 2A).^[3]^

#### 3.1.2. Septic arthritis (unilateral case).

Septic arthritis is usually associated with head and neck infections, and occasionally with hematogenous dissemination.^[16]^ It causes malocclusion, trismus, pain, swelling, fever, regional lymphadenopathy, fibrosis or ankylosis, and abnormal growth.^[16]^*Staphylococcus aureus* is the most common pathogen in this condition.^[16]^

#### 3.1.3. Condylar hyperplasia (unilateral case).

Condylar hyperplasia is a progressive and pathologic overgrowth of the mandibular condyle that can cause malocclusion and dentofacial deformity.^[17,18]^ It is associated with neoplasia, trauma, infection, abnormal condylar loading, hormonal influence, heredity, and aberrant growth factors.^[18]^ In 2014, Wolford et al suggested classification into 4 types based on clinical characteristics, imaging examination findings, and histology: Type 1 is an accelerated and prolonged aberration of normal condylar growth; Type 2 is included in condylar pathologic entities such as osteochondroma, Type 3 is included in other types of benign tumors, and Type 4 is included in malignant tumors arising from the mandibular condyle.^[17,18]^ Thus, Type 1 is a self-limiting condition, whereas the others do not.^[17,18]^

#### 3.1.4. Dislocation (unilateral case).

Anterior dislocation commonly presents with characteristic clinical symptoms such as pain, tenderness, mouth-closing disability, speech disability, salivation, and facial deformity.^[19]^

### 3.2. Posterior open bite on the unaffected side and/or mandibular shift toward the affected side

#### 3.2.1. TMD (unilateral case).

The TMJ condition of disc displacement without reduction causes reduced disc space and leads to heavy contact on the affected side and an open bite on the unaffected side (Fig. 2B).^[3]^

#### 3.2.2. Idiopathic condylar resorption (unilateral case).

Idiopathic condylar resorption is a progressive degenerative disease of the TMJ, without specific local or systemic etiologies.^[20–22]^ It can cause malocclusion, dentofacial deformity, and TMJ symptoms. However, 25% of the patients had no TMJ symptoms.^[20–22]^

#### 3.2.3. Juvenile idiopathic arthritis (unilateral case).

Juvenile idiopathic arthritis is the most common type of arthritis in children under 16 years of age.^[23,24]^ It was formerly known as juvenile rheumatoid arthritis, juvenile chronic arthritis, Still disease, or juvenile chronic polyarthritis.^[25]^ Inflammation occurs in ≥1 joints, and almost half of the patients have at least one sign or symptom attributable to TMJ.^[26,27]^ Imaging examination of the TMJ reveals condylar bony changes, which lead to disturbances in dentofacial growth and mandibular function.^[23–25]^ It causes anterior and/or lateral open bite, trismus, decreased chewing ability, jaw and facial pain, headache, craniofacial abnormality, and jaw opening limitation.^[27]^

#### 3.2.4. Rheumatoid arthritis (unilateral case).

Rheumatoid arthritis involvement in the TMJ causes malocclusion and pain, and inflammation occurs in ≥1 joints.^[26]^ Imaging examinations reveal condylar bony changes, which occasionally progress to ankylosis.^[26]^

#### 3.2.5. Condylar fracture (unilateral case).

Condylar fracture usually relates to various accidents.^[28–30]^ It accounts for 9% to 49% of all mandibular fractures and causes trismus, pain, and malocclusion.^[28–33]^ It is associated with fibrous or bony ankylosis.^[31]^

#### 3.2.6. Articular disc fracture (unilateral case).

This term indicates complete separation of the TMJ disc.^[34]^ It can cause mild, dull TMJ pain, and acute malocclusion, including vertical and/or lateral occlusal changes followed by mastication difficulty.^[34]^

#### 3.2.7. Ankylosis (unilateral case).

Ankylosis is divided into 3 conditions: intra-articular or extra-articular, bony/fibrous, or fibro-osseous, complete, or incomplete.^[35–37]^ It causes malocclusion, trismus, and craniofacial abnormalities.^[35–37]^ It is associated with trauma, local or systemic infection, and systemic diseases such as ankylosing spondylitis, rheumatoid arthritis, and psoriasis.^[35–37]^

#### 3.2.8. Dislocation (unilateral case).

There are rare cases of fracturing of the glenoid fossa with mandibular condyle dislocation into the middle cranial fossa without mandibular condyle fractures, namely upward dislocation.^[38]^ This can cause malocclusion, pain, trismus, facial asymmetry, cerebrospinal fluid leak, laceration of the external auditory canal, and various neurological findings.^[38]^

### 3.3. Bilateral posterior open bite

#### 3.3.1. Condylar hyperplasia (bilateral case).

It is possible that type 1 condylar hyperplasia occurs bilaterally (Fig. 2C).^[17,18]^

### 3.4. Anterior open bite and/or clockwise rotation of the mandible

#### 3.4.1. TMD (bilateral case).

TMD is associated with reduced mandibular ramus height, causing a clockwise rotation of the mandibular plane (Fig. 2D).^[39]^

#### 3.4.2. Idiopathic condylar resorption (bilateral case).

Bilateral idiopathic condylar resorption causes malocclusion with or without an anterior open bite.^[20–22]^

#### 3.4.3. Juvenile idiopathic arthritis (bilateral case).

Juvenile idiopathic arthritis usually occurs bilaterally.^[23–27]^

#### 3.4.4. Rheumatoid arthritis (bilateral case).

Bony changes related to rheumatoid arthritis generally occur bilaterally.^[26]^

#### 3.4.5. Condylar fracture (bilateral case).

Bilateral condylar fractures cause an anterior open bite with clockwise rotation of the mandibular plane.^[28–33]^

#### 3.4.6. Articular disc fracture (bilateral case).

Bilateral cases cause an anterior open bite with clockwise rotation of the mandible.^[34]^

#### 3.4.7. Ankylosis (bilateral case).

Bilateral ankylosis causes anterior open bite with clockwise rotation of the mandibular plane, limitation of jaw opening, and TMJ pain.^[35–37]^

#### 3.4.8. Dislocation (bilateral case).

Bilateral dislocation causes an anterior open bite with clockwise rotation of the mandibular plane, with pain and mouth closing disturbance.^[19]^

### 3.5. Others

#### 3.5.1. Chromosomal and genetic abnormalities or congenital anomalies.

Hemifacial microsomia is characterized by facial asymmetry associated with bone deficiency, hypoplasia, or the absence of soft tissue.^[40]^ Malocclusion was observed with these features.^[40]^

Auriculocondylar syndrome is characterized by prominent malformed ear, condylar aplasia or hypoplasia, and a number of other features associated with auricular and oral abnormalities.^[41]^ Malocclusion was observed with such features.^[41]^

Hallermann-Streiff syndrome is characterized by dyscephaly, short stature, bilateral microphthalmia, cataracts, hypotrichosis, and skin atrophy.^[42]^ Dental abnormalities, such as malocclusion, severe caries, enamel hypoplasia, supernumerary and neonatal teeth, hypodontia, and premature eruption of primary teeth, are present in 80% of patients with this syndrome.^[42]^

## 4. Discussions

In the Diagnostic Criteria for Temporomandibular Disorders, imaging examinations were not required for the initial diagnosis of TMD. Clinical diagnoses were derived from information such as clinical characteristics and symptoms.^[43]^ However, medical and dental clinicians who treat malocclusion must have clinical knowledge about the relationship between TMJ diseases and characteristics, including malocclusion. However, there are no reported reviews summarizing and discussing them.

This literature review was conducted to summarize clinical knowledge, including malocclusion, described in the published literature on TMJ diseases, including TMD. The authors also set out to create a diagnostic tree of TMJ diseases based on the types of malocclusion and the clinical characteristics and symptoms of the 4 variables. This study covers malocclusion cases that were not only conscious occlusal problems, but also identifiable and obvious ones. Thus, cases of subjective occlusal symptoms without obvious identifiable occlusal disharmony, such “phantom bite syndrome” and “occlusal discomfort syndrome,” were excluded.^[44,45]^ To the best of the authors’ knowledge, this is the first literature review, and the current study suggests that it is important to diagnose TMJ diseases based on clinical characteristics and symptoms, including malocclusion. The authors thought that summarizing clinical knowledge of TMJ diseases and the diagnostic tree suggested in this study might play an important role in the differential diagnosis of TMJ diseases related to malocclusion in clinical practice. Furthermore, the authors believe that these can help medical and dental clinicians treat malocclusion to avoid overlooking causative diseases. This is thought to be a breakthrough in this specialized area.

The authors suggest that malocclusion can be categorized into 5 types: posterior open bite on the affected side and/or mandibular shift toward the unaffected side; posterior open bite on the unaffected side and/or mandibular shift toward the affected side, bilateral posterior open bite, anterior open bite and/or clockwise rotation of the mandible, and others. In addition, the authors proposed a differential diagnostic method for TMJ diseases based on 4 clinical characteristics and symptoms: type of malocclusion, acute or chronic occlusal change, presence or absence of pain, and presence or absence of limitation of mouth opening. The authors believe that the 5 types of malocclusion and the 4 clinical characteristics and symptoms provide useful information for the differential diagnosis of TMJ diseases.

Some diseases and conditions related to malocclusion, such as septic arthritis, type 4 condylar hyperplasia, and condylar dislocation into the middle cranial fossa, may lead to serious conditions, including death.^[14,17,18,38,46–49]^ Thus, it is important to diagnose temporomandibular joint diseases as soon as possible, and to prevent delay of treatment. TMD is widely accepted as a self-limiting condition. TMD signs and symptoms usually show a natural tendency to improve.^[50]^ Thus, conservative therapy is the most common treatment for TMD. Malocclusions resulting from inflammatory reactions, including those associated with joint effusion, may improve naturally. However, the authors reported that malocclusions associated with disc dislocation are occasionally observed, and these may not improve naturally.^[13]^ To make matters worse, they tend to appear in the chronic phase, in which mouth opening limitation improves.^[13]^ Therefore, the authors suggest that it is important to make proper differential diagnosis at first so as not to overlook the causative disease related to malocclusion, and recommend that clinicians set an appropriate follow-up endpoint considering the possibility of occlusal changes after improvement in TMJ diseases.

Malocclusion cases may include congenital, progressive, or serious diseases and conditions. Additionally, tooth decay, periapical lesions, and periodontal disease of deciduous and permanent teeth can occur during malocclusion. Therefore, specialists, including medical doctors, general dentists, orthodontists, oral and maxillofacial surgeons, and speech-language-hearing therapists must be involved in managing these conditions and in maintaining, restoring, and/or improving quality of life.

Regarding the limitations of this study, few studies have discussed the association between TMJ diseases and malocclusion. The authors only included 28 relevant published works in this study. Additionally, because there is little literature on malocclusion associated with TMJ diseases in humans, this review was conducted based on case reports, case series, and narrative reviews. The authors believe that further reports describing detailed information and analysis of associations between TMJ disease and malocclusion are required to avoid duplicate categorization of TMJ diseases into 5 types of malocclusion in this study. Therefore, the diagnostic tree has room for improvement. In the future, further clinical knowledge of the associations between malocclusion and causative TMJ diseases should be accumulated, and the diagnostic tree should be updated based on new information.

## 5. Conclusions

Clinicians treating malocclusion need to try differential diagnosis at first, and it is important that they do not overlook causative diseases. This study reviewed the relationship between malocclusion and causative TMJ diseases. The summarized information and preliminary diagnostic tree suggested in this study will be helpful for the differential diagnosis of causative TMJ disease in the future.

## Author contributions

Conceptualization: Shinpei Matsuda

Data curation: Shinpei Matsuda

Visualization: Saki Mikami, Shinpei Matsuda, Taihiko Yamaguchi

Writing – original draft: Shinpei Matsuda, Taihiko Yamaguchi

Writing – review & editing: Akihito Gotouda, Hitoshi Yoshimura, Saki Mikami, Shinpei Matsuda, Taihiko Yamaguchi

## References

[1] Shen L He F Zhang C. Prevalence of malocclusion in primary dentition in mainland China, 1988–2017: a systematic review and meta-analysis. Sci Rep 2018;8:4716. 29549346 10.1038/s41598-018-22900-x PMC5856803

[2] Zou J Meng M Law CS. Common dental diseases in children and malocclusion. Int J Oral Sci 2018;10:7. 29540669 10.1038/s41368-018-0012-3 PMC5944594

[3] Dupont JS. Acute malocclusion. Gen Dent 2006;54:102–4. 16689064

[4] Tak M Nagarajappa R Sharda AJ. Prevalence of malocclusion and orthodontic treatment needs among 12-15 years old school children of Udaipur, India. Eur J Dent 2013;7 (suppl 1):S45–53. 10.4103/1305-7456.119071 PMC4054079 24966728

[5] Shroff B. Malocclusion as a cause for temporomandibular disorders and orthodontics as a treatment. Oral Maxillofac Surg Clin North Am 2018;30:299–302. 29866453 10.1016/j.coms.2018.04.006

[6] Marinho LH McLoughlin PM. Lateral open bite resulting from acute temporomandibular joint effusion. Br J Oral Maxillofac Surg 1994;32:127–8. 8199147 10.1016/0266-4356(94)90146-5

[7] Brustowicz KA Padwa BL. Malocclusion in children caused by temporomandibular joint effusion. Int J Oral Maxillofac Surg 2013;42:1034–6. 23623725 10.1016/j.ijom.2013.03.017

[8] Scrivani SJ Keith DA Kaban LB. Temporomandibular disorders. N Engl J Med 2008;359:2693–705. 19092154 10.1056/NEJMra0802472

[9] Türp JC Schindler H. The dental occlusion as a suspected cause for TMDs: epidemiological and etiological considerations. J Oral Rehabil 2012;39:502–12. 22486535 10.1111/j.1365-2842.2012.02304.x

[10] Xie Q Li X Xu X. The difficult relationship between occlusal interferences and temporomandibular disorder - insights from animal and human experimental studies. J Oral Rehabil 2013;40:279–95. 23356664 10.1111/joor.12034

[11] Manfredini D Lombardo L Siciliani G. Temporomandibular disorders and dental occlusion. A systematic review of association studies: end of an era? J Oral Rehabil 2017;44:908–23. 28600812 10.1111/joor.12531

[12] Chen YJ Shih TT Wang JS. Magnetic resonance images of the temporomandibular joints of patients with acquired open bite. Oral Surg Oral Med Oral Pathol Oral Radiol Endod 2005;99:734–42. 15897861 10.1016/j.tripleo.2004.10.020

[13] Gotouda A Yamaguchi T Kaneko T. Clinical characteristics of acquired anterior open bite subsequent to TMJ closed lock. Ann Jpn Prosthodont Soc 2016;8:281–8. [Japanese (abstract in English)].

[14] Matsuda S Yoshimura H Kondo S. Temporomandibular dislocation caused by pancreatic cancer metastasis: a case report. Oncol Lett 2017;14:6053–8. 29113245 10.3892/ol.2017.6951 PMC5661556

[15] Matsuda S Yoshimura H Hamano T. Post-traumatic trigeminal neuropathy caused by an orbital stab wound. J Craniofac Surg 2017;28:e28–30. 27875513 10.1097/SCS.0000000000003175

[16] Ayachi S Mziou Z Moatemri R. Bilateral septic arthritis of the temporomandibular joint: case report. Pan Afr Med J 2016;25:100. 28292063 10.11604/pamj.2016.25.100.7943 PMC5325521

[17] Wolford LM Movahed R Dhameja A. Low condylectomy and orthognathic surgery to treat mandibular condylar osteochondroma: a retrospective review of 37 cases. J Oral Maxillofac Surg 2014;72:1704–28. 24997022 10.1016/j.joms.2014.03.009

[18] Rodrigues DB Castro V. Condylar hyperplasia of the temporomandibular joint: types, treatment, and surgical implications. Oral Maxillofac Surg Clin North Am 2015;27:155–67. 25483450 10.1016/j.coms.2014.09.011

[19] Nakashima M Yano H Akita S. Traumatic unilateral temporomandibular joint dislocation overlooked for more than two decades. J Craniofac Surg 2007;18:1466–70. 17993903 10.1097/scs.0b013e31814fb5af

[20] Wang J Veiszenbacher E Waite PD. Comprehensive treatment approach for bilateral idiopathic condylar resorption and anterior open bite with customized lingual braces and total joint prostheses. Am J Orthod Dentofacial Orthop 2019;156:125–36. 31256825 10.1016/j.ajodo.2018.02.017

[21] Wolford LM Cardenas L. Idiopathic condylar resorption: diagnosis, treatment protocol, and outcomes. Am J Orthod Dentofacial Orthop 1999;116:667–77. 10587602 10.1016/s0889-5406(99)70203-9

[22] Kajii TS Fujita T Sakaguchi Y. Osseous changes of the mandibular condyle affect backward-rotation of the mandibular ramus in Angle Class II orthodontic patients with idiopathic condylar resorption of the temporomandibular joint. Cranio 2019;37:264–71. 29359644 10.1080/08869634.2017.1421446

[23] Ronchezel MV Hilário MO Goldenberg J. Temporomandibular joint and mandibular growth alterations in patients with juvenile rheumatoid arthritis. J Rheumatol 1995;22:1956–61. 8991998

[24] Kjellberg H. Juvenile chronic arthritis. Dentofacial morphology, growth, mandibular function and orthodontic treatment. Swed Dent J Suppl 1995;109:1–56. 7660317

[25] Kjellberg H. Craniofacial growth in juvenile chronic arthritis. Acta Odontol Scand 1998;56:360–5. 10066117 10.1080/000163598428329

[26] Marbach JJ. Arthritis of the temporomandibular joints. Am Fam Physician 1979;19:131–9. 760437

[27] Meyers AB Laor T. Magnetic resonance imaging of the temporomandibular joint in children with juvenile idiopathic arthritis. Pediatr Radiol 2013;43:1632–41. 24257698 10.1007/s00247-013-2769-z

[28] Hovinga J Boering G Stegenga B. Long-term results of nonsurgical management of condylar fractures in children. Int J Oral Maxillofac Surg 1999;28:429–40. 10609744

[29] Smets LM Van Damme PA Stoelinga PJ. Non-surgical treatment of condylar fractures in adults: a retrospective analysis. J Craniomaxillofac Surg 2003;31:162–7. 12818602 10.1016/s1010-5182(03)00025-8

[30] Becking AG Zijderveld SA Tuinzing DB. The surgical management of post-traumatic malocclusion. Clin Plast Surg 2007;34:e37–43. 17692694 10.1016/j.cps.2007.04.007

[31] Chen M Yang C He D. Soft tissue reduction during open treatment of intracapsular condylar fracture of the temporomandibular joint: our institution's experience. J Oral Maxillofac Surg 2010;68:2189–95. 20576338 10.1016/j.joms.2009.09.063

[32] Kokemueller H Konstantinovic VS Barth EL. Endoscope-assisted transoral reduction and internal fixation versus closed treatment of mandibular condylar process fractures--a prospective double-center study. J Oral Maxillofac Surg 2012;70:384–95. 21664746 10.1016/j.joms.2011.02.035

[33] Gvenetadze Z Danelia T Nemsadze G. Topical diagnostics of traumatic condylar injuries and alloplastic reconstruction of temporomandibular joint heads. Georgian Med News 2014;229:10–5. 24850598

[34] An SY Jung JK. Fracture of the articular disc in the temporomandibular joint: two case reports. Dentomaxillofac Radiol 2015;44:20140218. 25308829 10.1259/dmfr.20140218 PMC4614171

[35] Akama MK Guthua S Chindia ML. Management of bilateral temporomandibular joint ankylosis in children: case report. East Afr Med J 2009;86:45–8. 19530549 10.4314/eamj.v86i1.46930

[36] Abdelrahman TF Takahashi K Bessho K. Posttraumatic temporomandibular joint ankylosis in adults: is it mandatory to perform interposition arthroplasty? J Craniofac Surg 2010;21:1301–4. 20647844 10.1097/SCS.0b013e3181e1e69a

[37] Babu L Jain MK Ramesh C. Is aggressive gap arthroplasty essential in the management of temporomandibular joint ankylosis?-a prospective clinical study of 15 cases. Br J Oral Maxillofac Surg 2013;51:473–8. 23219020 10.1016/j.bjoms.2012.11.004

[38] Oberman B Setabutr D Goldenberg D. Traumatic dislocation of intact mandibular condyle into middle cranial fossa. Am J Otolaryngol 2014;35:251–3. 24462110 10.1016/j.amjoto.2013.11.004

[39] Shi JJ Zhang F Zhou YQ. The relationship between partial disc displacement and mandibular dysplasia in female adolescents. Med Sci Monit 2010;16:CR283–8. 20512091

[40] Munro IR Phillips JH Griffin G. Growth after construction of the temporomandibular joint in children with hemifacial microsomia. Cleft Palate J 1989;26:303–11. 2805348

[41] Storm AL Johnson JM Lammer E. Auriculo-condylar syndrome is associated with highly variable ear and mandibular defects in multiple kindreds. Am J Med Genet A 2005;138A:141–5. 16114046 10.1002/ajmg.a.30883

[42] Tuna EB Sulun T Rosti O. Craniodentofacial manifestations in Hallermann-Streiff syndrome. Cranio 2009;27:33–8. 19241797 10.1179/crn.2009.006

[43] Schiffman E Ohrbach R Truelove E. Diagnostic Criteria for Temporomandibular Disorders (DC/TMD) for clinical and research applications: recommendations of the international RDC/TMD consortium network* and orofacial pain special interest group†. J Oral Facial Pain Headache 2014;28:6–27. 24482784 10.11607/jop.1151 PMC4478082

[44] Marbach JJ. Phantom bite syndrome. Am J Psychiatry 1978;135:476–9. 637145 10.1176/ajp.135.4.476

[45] Tamaki K Ishigaki S Ogawa T. Japan Prosthodontic Society position paper on “occlusal discomfort syndrome. J Prosthodont Res 2016;60:156–66. 26868189 10.1016/j.jpor.2015.11.002

[46] Dias Ferraz A Spagnol G Alves Maciel F. Septic arthritis of the temporomandibular joint: case series and literature review. Cranio 2019;39:541–8. 31478470 10.1080/08869634.2019.1661943

[47] Cai XY Yang C Zhang ZY. Septic arthritis of the temporomandibular joint: a retrospective review of 40 cases. J Oral Maxillofac Surg 2010;68:731–8. 19954877 10.1016/j.joms.2009.07.060

[48] Wolford LM Movahed R Perez DE. A classification system for conditions causing condylar hyperplasia. J Oral Maxillofac Surg 2014;72:567–95. 24388179 10.1016/j.joms.2013.09.002

[49] Smolka W Brekenfeld C Büchel P. Metastatic adenocarcinoma of the temporomandibular joint from the cardia of the stomach: a case report. Int J Oral Maxillofac Surg 2004;33:713–5. 15337187 10.1016/j.ijom.2003.10.013

[50] Yatani H Kaneshima T Kuboki T. Long-term follow-up study on drop-out TMD patients with self-administered questionnaires. J Orofac Pain 1997;11:258–69. 9610316

